# Evidence for the Presence of Glucosensor Mechanisms Not Dependent on Glucokinase in Hypothalamus and Hindbrain of Rainbow Trout (*Oncorhynchus mykiss*)

**DOI:** 10.1371/journal.pone.0128603

**Published:** 2015-05-21

**Authors:** Cristina Otero-Rodiño, Marta Librán-Pérez, Cristina Velasco, Marcos A. López-Patiño, Jesús M. Míguez, José L. Soengas

**Affiliations:** Laboratorio de Fisioloxía Animal, Departamento de Bioloxía Funcional e Ciencias da Saúde, Facultade de Bioloxía, Universidade de Vigo, Vigo, Spain; Universitat de Barcelona, SPAIN

## Abstract

We hypothesize that glucosensor mechanisms other than that mediated by glucokinase (GK) operate in hypothalamus and hindbrain of the carnivorous fish species rainbow trout and stress affected them. Therefore, we evaluated in these areas changes in parameters which could be related to putative glucosensor mechanisms based on liver X receptor (LXR), mitochondrial activity, sweet taste receptor, and sodium/glucose co-transporter 1 (SGLT-1) 6h after intraperitoneal injection of 5 mL.Kg^-1^ of saline solution alone (normoglycaemic treatment) or containing insulin (hypoglycaemic treatment, 4 mg bovine insulin.Kg^-1^ body mass), or D-glucose (hyperglycaemic treatment, 500 mg.Kg^-1^ body mass). Half of tanks were kept at a 10 Kg fish mass.m^-3^ and denoted as fish under normal stocking density (NSD) whereas the remaining tanks were kept at a stressful high stocking density (70 kg fish mass.m^-3^) denoted as HSD. The results obtained in non-stressed rainbow trout provide evidence, for the first time in fish, that manipulation of glucose levels induce changes in parameters which could be related to putative glucosensor systems based on LXR, mitochondrial activity and sweet taste receptor in hypothalamus, and a system based on SGLT-1 in hindbrain. Stress altered the response of parameters related to these systems to changes in glycaemia.

## Introduction

Glucosensor mechanisms allow vertebrates to monitor changes in glucose levels at different central or peripheral locations [1]. Glucosensing in brain areas like hypothalamus and hindbrain has been related to the control of food intake as well as to counter-regulatory mechanisms to restore plasma levels of metabolites [1]. The mechanisms involved in glucosensing have been partially elucidated in mammals [1,2]. The most important and best characterized mechanism is that demonstrated in pancreatic β-cells and glucose-excited (GE) neurons dependent on glucokinase (GK), glucose facilitative transporter type 2 (GLUT2), and ATP-dependent inward rectifier potassium channel (K_ATP_) [1]. However, since not all the glucosensing neurons rely on this mechanism [3] the existence of alternative glucosensing mechanisms has been suggested. Thus, evidence has been obtained in recent years in different peripheral and central areas of mammals, specially in omnivorous species like dog and rodents. Glucose is a direct agonist of liver X receptor (LXR) [4] whose expression is stimulated by high glucose concentrations resulting in an inhibition of gluconeogenesis [5,6]. The stimulation by glucose of sweet taste receptors (similar to those described in lingual taste cells) depending on a heterodimer of type 1 taste receptor subunits (T1Rs) formed by T1R2+T1R3 and α-gustducin, a transducin-like heterotrimeric G protein, activates an intracellular signaling cascade [7]. The expression of sodium/glucose co-transporter 1 (SGLT-1) increases in response to enhanced glucose levels in tissues like intestine [8] and brain [9,10]. Finally, another metabolism-dependent but ATP-independent mechanism suggested to contribute to hypothalamic nutrient sensing relies on mitochondrial production of reactive oxygen species (ROS) by electron leakage during intracellular glucose and fatty acid (FA) metabolism [2] leading to increased expression of uncoupling protein 2 (UCP2). In addition, several of these systems appear to be inter-connected. Thus, for instance, T1R3 and α-gustducin are necessary for increased SGLT-1 induction by dietary carbohydrates [11]. Few studies have been carried out in carnivorous mammalian species like cat regarding the presence and functioning of alternative glucosensor mechanisms, and only peripheral (but not central) areas like intestine [12] were assessed.

Glucose is not the main energy substrate of carnivorous fish species, which rely more on protein and lipid for energy purposes [13,14]. However, since glucose metabolism is important for the functioning of specific tissues like the brain [13], fish do control circulating glucose levels [1,15]. Accordingly, we have demonstrated the presence in brain areas (hypothalamus and hindbrain) of a carnivorous fish species like rainbow trout (*Oncorhynchus mykiss*) of a glucosensor mechanism based on GK-GLUT2-K_ATP_ similar to that characterized in mammalian GE neurons and pancreatic β-cells, which is related to the control of food intake, and to counter-regulatory mechanisms [1,13,14,15]. As for alternative glucosensor mechanisms, there is evidence obtained in fish concerning the presence of several of their components. Thus, i) orthologs of mammalian LXR are expressed in rainbow trout and Atlantic salmon tissues including brain [16,17]; ii) LXR agonists induce metabolic changes in zebrafish liver [18] and Atlantic salmon SHK-1 cells [19]; iii) UCP2 is expressed ubiquitously in gilthead sea bream [20], sea bass [21] and rainbow trout [22,23]; iv) T1Rs are present in tissues of rainbow trout [24]; and, v) SGLT-1 is present in different tissues of rainbow trout [17,24,25,26]. Moreover, in preliminary studies carried out in rainbow trout, we demonstrated the presence of components of putative glucosensor mechanisms other than that mediated by GK in peripheral tissues such as intestine [24,27] and head kidney [26]. In head kidney neither SGLT-1, Gnat3 (gene expressing α-gustducin) or LXR mRNA abundance were affected by changes in glucose concentration [26]. In contrast, in intestine these parameters displayed changes that were dependent either *in vivo* or *in vitro* on changes in glucose levels [24]. To date, there is no available information in fish, or in any other non-mammalian vertebrate, regarding the hypothetical presence and functioning in brain areas of alternative glucosensor mechanisms. Based on our previous studies in rainbow trout, we hypothesize that alternative glucosensing mechanisms are present in hypothalamus and hindbrain responding to changes in circulating levels of glucose.

Therefore, *we aimed to evaluate in* hypothalamus and hindbrain *of rainbow trout the presence and response to changes in glucose levels* of parameters which could be related to putative alternative glucosensor mechanisms based on: i) LXR, such as fructose 1,6-bisphosphatase (FBPase) and phospho(enol)pyruvate carboxykinase (PEPCK) activities, and mRNA abundance of liver X receptor α (LXRα), FBPase, PEPCK, peroxisome proliferator-activated receptor type γ (PPARγ), and sterol regulatory element-binding protein type 1c (SREBP1c); ii) mitochondrial activity, such as carnitine palmitoyltransferase 1 (CPT-1) and hydroxyacil-CoA dehydrogenase (HOAD) activities, and mRNA abundance of CPT-1c, CPT-1d, HOAD, cytochrome c oxidase subunit 4 (COX4), and UCP2a; iii) sweet taste receptor, such as mRNA abundance of type 1 taste receptor subunit 2 (T1R2), type 1 taste receptor subunit 3 (T1R3), and α-gustducin (Gnat3); and, iv) SGLT-1 by assessing its mRNA abundance. As a positive control, we also evaluated changes in parameters related to GK-mediated glucosensing, such as GK and pyruvate kinase (PK) activities, and mRNA abundance of GK, GLUT2, PK, 6-phosphofructo 1-kinase (PFK), inward rectifier K^+^ channel pore type 6.x-like (Kir6.x-like), and sulfonylurea receptor-like (SUR-like), as well as in the mRNA abundance of neuropeptides related to the control of food intake, such as neuropeptide Y (NPY), pro-opio melanocortin A1 (POMC-A1), cocaine and amphetamine-related transcript (CART), agouti-related peptide (AgRP), and cortocotropin releasing factor (CRF).

We previously demonstrated in rainbow trout that the response of the glucosensor system based on GK is modified under stress conditions [28,29], which are also characterized by increased glycaemia, in a way that the glucosensing system is not properly informing about changes in circulating glucose levels. We therefore also aimed to evaluate if the response of putative alternative glucosensor mechanisms would be also altered under the stress conditions elicited by high stocking density.

## Materials and Methods

### Ethics statement

The experiments described comply with the Guidelines of the European Union Council (2010/63/UE), and of the Spanish Government (RD 55/2013) for the use of animals in research. The Ethics Committee of the Universidade de Vigo approved the procedures.

### Fish

Rainbow trout obtained from a local fish farm (A Estrada, Spain) were maintained for 1 month in 12 100L-tanks (2 replicate tanks per group containing 7–8 fish each) under laboratory conditions, 12L:12D photoperiod, and dechlorinated tap water at 15°C. Fish weight was 99 ± 3 g. Trout were fed once daily to satiety with commercial dry fish pellets (Dibaq-Diproteg SA, Spain; proximate food analysis was 48% crude protein, 14% carbohydrates, 25% crude fat, and 11.5% ash; and 20.2 MJ/kg of feed).

### Experimental design

Following acclimation, fish were fasted for 24h before treatment to ensure fish had basal hormone levels. On the day of experiment, fish were anaesthetized by the addition of 2-phenoxyethanol (Sigma Chemical Co., St Louis, MO, USA; 0.2% v/v) to each tank, and weighed. Then, 15 fish per group (coming from two replicate tanks containing 7–8 fish per tank) received intraperitoneally (IP) 5 mL.Kg^-1^ injection of saline solution alone (normoglycaemic treatment) or containing insulin (hypoglycaemic treatment, 4 mg bovine insulin.Kg^-1^ body mass, insulin from Sigma Chemical), or D-glucose (hyperglycaemic treatment, 500 mg.Kg^-1^ body mass). Immediately after injection, fish returned to anaesthetic-free tanks where remained for 6h without any access to food. Six tanks (two tank replicates per glycaemic treatment) were kept at 10 Kg fish mass.m^-3^ and denoted as fish under normal stocking density (NSD). In the remaining 6 tanks (two tank replicates per glycaemic treatment) a quantity of water was removed until reaching a stressful high stocking density (70 kg fish mass.m^-3^) denoted as HSD. Therefore, the 6 experimental groups used (2 tank replicates with 7–8 fish, N = 15) were maintained for 6h under different glycaemic conditions, and under different stocking densities as follows: 1) normoglycaemic fish under NSD, 2) hypoglycaemic fish under NSD, 3) hyperglycaemic fish under NSD, 4) normoglycaemic fish under HSD, 5) hypoglycaemic fish under HSD, and 6) hyperglycaemic fish under HSD. In each group, 10 fish were used to assess enzyme activities and metabolite levels whereas the remaining 5 fish were sampled for the assessment of mRNA levels by q-PCR. In each sampling, fish were anesthesized as above, and blood was collected from the caudal vein with a heparinised syringe. Fish were sacrificed by decapitation, and hypothalamus and hindbrain were taken, frozen, and stored as previously described [30,31,32,33,34].

### Assessment of metabolite levels and enzyme activities

Levels of glucose in plasma were determined enzymatically using a commercial kit (Biomérieux, Grenoble, France) adapted to a microplate format. Plasma cortisol levels were assessed by ELISA using a commercially available kit (Cayman, Ann Harbor, MI, USA).

Samples used to assess metabolite levels in hypothalamus and hindbrain were homogenized immediately by ultrasonic disruption in 7.5 vols of ice-cooled 0.6 M perchloric acid, and neutralized (using 1 M potassium bicarbonate). The homogenate was centrifuged (10,000 g), and the supernatant used to assay tissue metabolites. Tissue glycogen levels were assessed using the method of Keppler and Decker [35]. Glucose obtained after glycogen breakdown (after subtracting free glucose levels) was determined with a commercial kit (Biomérieux).

Samples for enzyme activities were homogenized by ultrasonic disruption in 10 vol ice-cold phosphorylation-dephosphorylation stopping buffer containing: 50 mM imidazole-HCl (pH 7.6), 15 mM 2-mercaptoethanol, 100 mM KF, 5 mM EDTA, 5 mM EGTA, and a protease inhibitor cocktail (Sigma, P-2714). The homogenate was centrifuged (10,000 g) and the supernatant used immediately for enzyme assays. Enzyme activities were determined using a microplate reader INFINITE 200 Pro (Tecan) and microplates. Reaction rates of enzymes were determined by the increase or decrease in absorbance of NAD(P)H at 340 nm or, in the case of CPT-1 activity, of 5,5’-Dithiobis(2-nitrobenzoic acid)-CoA complex at 412 nm. The reactions were started by the addition of supernatant (15 μl) at a pre-established protein concentration, omitting the substrate in control wells (final volume 265–295 μl), and allowing the reactions to proceed at 20°C for pre-established time periods. Enzyme activities are expressed per protein level, which was assayed according to the bicinchoninic acid method with bovine serum albumin (Sigma) as standard. Enzyme activities were assessed at maximum rates determined by preliminary tests to determine optimal substrate concentrations. CPT-1 (*EC* 2.3.1.21), FBPase (*EC* 3.1.3.11), GK (*EC* 2.7.1.2), HOAD (*EC* 1.1.1.35), PEPCK (*EC* 4.1.1.32), and PK (*EC*. 2.7.1.40) activities were determined as described previously [30,31,32,33,34,36].

### mRNA abundance analysis by real-time quantitative RT-PCR

Total RNA extracted from tissues using Trizol reagent (Life Technologies, Grand Island, NY, USA) was treated with RQ1-DNAse (Promega, Madison, WI, USA). 2 μg total RNA were reverse transcribed into cDNA using Superscript II reverse transcriptase (Promega) and random hexaprimers (Promega). Gene expression levels were determined by real-time quantitative RT-PCR (q-PCR) using the iCycler iQ (BIO-RAD, Hercules, CA, USA). Analyses were performed using the MAXIMA SYBR Green qPCR Mastermix (Thermo Scientific, Waltham, MA, USA), in a total PCR reaction volume of 15 μl, containing 50–500 nM of each primer. mRNA abundance of transcripts (AgRP, CART, COX4, CPT-1, FBPase, GLUT2, GK, Gnat3, HOAD, Kir6.x-like, LXRα, NPY, PEPCK, PFK, PK, POMC-A1, PPARγ, SGLT-1, SREBP1c, SUR-like, T1R2, T1R3, and UCP2a) was determined as previously described in the same species [24,29,34,36,37,38,39,40,41,42,43,44,45,46]. Sequences and accession numbers of the primers used for each gene expression are shown in Table 1. Relative quantification of the target gene transcripts used β-actin gene as housekeeping (stably expressed throughout the experiment).

**Table 1:** Nucleotide sequences of the probes used to evaluate mRNA abundance qPCR.

	Forward primer	Reverse primer	Data base	Accession Number
β-Actin	GATGGGCCAGAAAGACAGCTA	TCGTCCCAGTTGGTGACGAT	GenBank	NM 001124235.1
AgRP	ACCAGCAGTCCTGTCTGGGTAA	AGTAGCAGATGGAGCCGAACA	GenBank	CR376289
CART	ACCATGGAGAGCTCCAG	GCGCACTGCTCTCCAA	GenBank	NM_001124627
COX4	TACGTGGGGGACATGGTGTT	CCCAGGAGCCCTTCTCCTTC	Sigenae	tcav0004c.i.22_3.1.s.om.8
CPT-1c	CGCTTCAAGAATGGGGTGAT	CAACCACCTGCTGTTTCTCA	GenBank	AJ619768
CPT-1d	CCGTTCCTAACAGAGGTGCT	ACACTCCGTAGCCATCGTCT	GenBank	AJ620356
CRF	ACAACGACTCAACTGAAGATCTCG	AGGAAATTGAGCTTCATGTCAGG	GenBank	AF296672
FBPase	GCTGGACCCTTCCATCGG	CGACATAACGCCCACCATAGG	GenBank	AF333188
GK	GCACGGCTGAGATGCTCTTTG	GCCTTGAACCCTTTGGTCCAG	GenBank	AF053331
GLUT2	GTGGAGAAGGAGGCGCAAGT	GCCACCGACACCATGGTAAA	GenBank	AF321816
Gnat3	GCAAGACGTGCTGAGGACCA	ATGGCGGTGACTCCCTCAAA	Sigenae	CU073912
HOAD	GGACAAAGTGGCACCAGCAC	GGGACGGGGTTGAAGAAGTG	Sigenae	tcad0001a.i.15 3.1.om
Kir6.x-like	TTGGCTCCTCTTCGCCATGT	AAAGCCGATGGTCACCTGGA	Sigenae	CA346261.1.s.om.8:1:773:1
LXRα	TGCAGCAGCCGTATGTGGA	GCGGCGGGAGCTTCTTGTC	GenBank	FJ470291
NPY	CTCGTCTGGACCTTTATATGC	GTTCATCATATCTGGACTGTG	GenBank	NM_001124266
PEPCK	GTTGGTGCTAAAGGGCACAC	CCCGTCTTCTGATAAGTCCAA	GenBank	AF246149
PFK	GGTGGAGATGCACAAGGAAT	CTTGATGTTGTCCCCTCCAT	Sigenae	tcbk0069c.k.05_s.1
PK	CCATCGTCGCGGTAACAAGA	ACATAGGAAAGGCCAGGGGC	GenBank	AF246146
POMC-A1	CTCGCTGTCAAGACCTCAACTCT	GAGTTGGGTTGGAGATGGACCTC	Tigr	TC86162
PPARγ	GACGGCGGGTCAGTACTTTA	ATGCTCTTGGCGAACTCTGT	DFCI	CA345564
SGLT-1	GGGCTGAACATCTACCTTGCT	CTCATAACCTCCCACCTCATTG	GenBank	AY210436
SREBP1c	GACAAGGTGGTCCAGTTGCT	CACACGTTAGTCCGCATCAC	GenBank	CA048941.1
SUR-like	CGAGGACTGGCCCCAGCA	GACTTTCCACTTCCTGTGCGTCC	Sigenae	tcce0019d.e.20_3.1.s.om.8
T1R2	GATGAGTGGGCCAGGAATGG	CCTCCCACCGGCTGACTTTA	Sigenae	FYV3OTN01AKLI0.s.om.10
T1R3	GCCCTGTGGAGCCCATCTTA	CCACACAGTAGGTCAGGGTGGA	Sigenae	GAY7CUQ01EHKNI.s.om.10
UCP2a	TCCGGCTACAGATCCAGG	CTCTCCACAGACCACGCA	GenBank	DQ295324

Agouti-related peptide (AgRP), cocaine and amphetamine-related transcript (CART), cytochrome c oxidase subunit 4 (COX4), carnitine palmitoyltransferase 1 (CPT-1), corticotrophin releasing factor (CRF), fructose 1,6-bisphosphatase (FBPase), glucokinase (GK), glucose facilitative transporter type 2 (GLUT2), α-gustducin (Gnat3), hydroxyacil-CoA dehydrogenase (HOAD), inward rectifier K^+^ channel pore type 6.x-like (Kir6.x-like), liver X receptor α (LXRα), neuropeptide Y (NPY), phospho(enol)pyruvate carboxykinase (PEPCK), 6-phosphofructo 1-kinase (PFK), pyruvate kinase (PK), pro-opio melanocortin A1 (POMC-A1), peroxisome proliferator-activated receptor type γ (PPARγ), sodium-glucose linked transporter type 1 (SGLT-1), sterol regulatory element-binding protein type 1c (SREBP1c), sulfonylurea receptor-like (SUR-like), type 1 taste receptor subunit 2 (T1R2), type 1 taste receptor subunit 3 (T1R3), and mitochondrial uncoupling protein 2a (UCP2a).

Thermal cycling was initiated by incubation at 95°C for 90s using hot-start iTaq DNA polymerase activation; 40 steps of PCR were performed, each one consisting of heating at 95°C for 15s for denaturing, annealing at specific temperatures for 30s, and extension at 72°C for 30s. Following the final PCR cycle, melting curves were systematically monitored (temperature gradient from 55 to 95°C) to ensure amplification of only one fragment. Each sample was assessed in triplicate. Samples without reverse transcriptase and samples without RNA were run for each reaction as negative controls. Only efficiency values between 85–100% were accepted (the R^2^ for all the genes assessed was always higher than 0.985). Relative quantification of the target gene transcript with the β-actin reference gene transcript was made following the Pfaffl method [47].

### Statistics

Comparisons among groups were carried out using two-way ANOVA with glycaemic treatment (hypo-, normo-, and hyper-glycaemic) and stocking density (normal and high) as main factors. When a significant effect was noted for a factor, a Student-Newman-Keuls test was used to assess significant (*P*<0.05) differences.

## Results

P-values obtained after two-way analysis of variance of results presented in the main text are shown in Table 2. P-values obtained after two-way analysis of variance of results presented in S1 Table.

**Table 2:** P-values obtained after two-way analysis of variance of parameters assessed in rainbow trout under different glycaemic conditions elicited by intraperitoneal (IP) administration of saline solution alone (normoglycaemic) or containing insulin (hypoglycaemic, 4 mg bovine insulin.Kg^-1^ body mass), or D-glucose (hyperglycaemic, 500 mg.Kg^-1^ body mass) kept at normal stocking density (NSD, 10 kg.m^-3^) or high stocking density (HSD, 70 kg.m^-3^) for 6 hours.

Parameter	Glycaemia	Stocking density	Glycaemia x stocking density
Plasma			
Glucose levels	0.001	0.001	-
Cortisol levels	-	0.001	-
Hypothalamus			
Glucose levels	0.001	-	-
Glycogen levels	0.034	-	0.006
LXRα mRNA abundance	0.044	-	0.036
FBPase activity	0.039	-	-
FBPase mRNA abundance	0.042	-	-
PEPCK mRNA abundance	0.031	-	0.007
PPARγ mRNA abundance	0.039	-	0.034
SREBP1c mRNA abundance	0.046	-	0.003
CPT-1 activity	0.038	-	0.025
CPT1c mRNA abundance	0.044	-	-
CPT1d mRNA abundance	0.040	-	-
HOAD activity	0.029	-	0.045
HOAD mRNA abundance	0.049	-	-
COX4 mRNA abundance	0.004	-	-
UCP2a mRNA abundance	0.011	-	0.045
T1R2 mRNA abundance	0.042	0.019	-
T1R3 mRNA abundance	0.015	-	0.048
Gnat3 mRNA abundance	0.016	-	-
SGLT-1 mRNA abundance	-	-	-
Hindbrain			
Glucose levels	0.001	-	0.030
Glycogen levels	0.001	-	-
LXRα mRNA abundance	0.017	-	-
FBPase activity	0.047	-	-
FBPase mRNA abundance	-	-	-
PEPCK activity	0.038	-	-
PEPCK mRNA abundance	0.010	-	-
PPARγ mRNA abundance	-	-	-
SREBP1c mRNA abundance	0.005	-	-
CPT-1 activity	0.046	-	-
CPT1c mRNA abundance	0.012	-	-
CPT1d mRNA abundance	-	-	-
HOAD activity	-	-	-
HOAD mRNA abundance	0.007	-	-
COX4 mRNA abundance	-	-	-
UCP2a mRNA abundance	0.001	-	-
T1R2 mRNA abundance	0.042	-	0.034
T1R3 mRNA abundance	0.039	-	-
Gnat3 mRNA abundance	-	-	-
SGLT-1 mRNA abundance	0.041	-	0.024

Glycaemia (hypo-, normo-, and hyper-) and stocking density (NSD and HSD) were the main factors. All values are significantly different unless noted by a dash.

**Fig 1:**
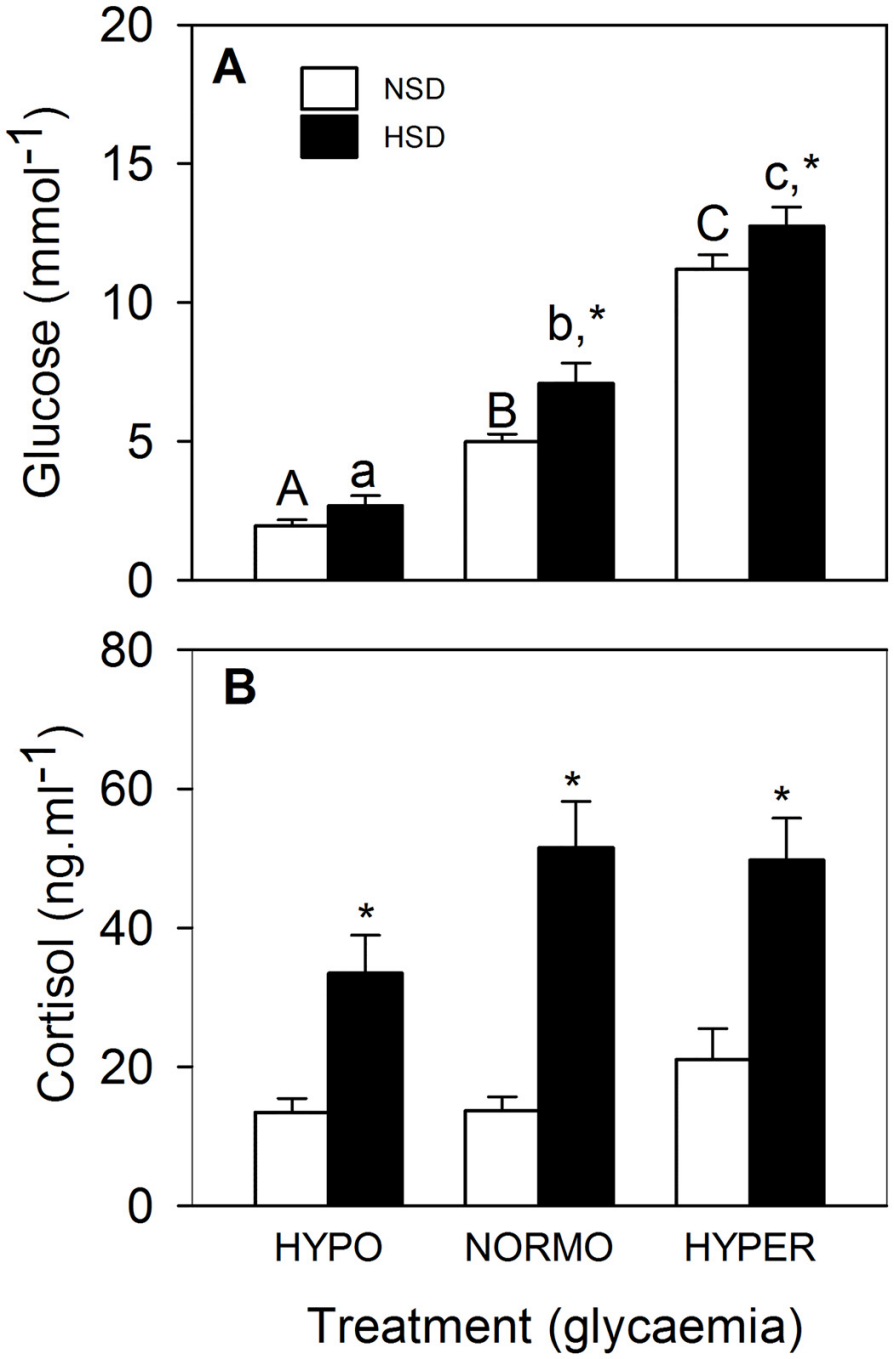
Levels of glucose (A) and cortisol (B) in plasma of rainbow trout under different glycaemic conditions elicited by intraperitoneal (IP) administration of saline solution alone (normoglycaemic) or containing insulin (hypoglycaemic, 4 mg bovine insulin.Kg^-1^ body mass), or D-glucose (hyperglycaemic, 500 mg.Kg^-1^ body mass) kept at normal stocking density (NSD, 10 kg.m^-3^) or high stocking density (HSD, 70 kg.m^-3^) for 6 hours. Data represent mean ± SEM of 10 measurements. *, significantly different (P<0.05) from NSD at the same glycaemic condition. Different letters indicate significant differences (P<0.05) from different glycaemic conditions at the same density (capital letters within NSD and lowercase letters within HSD).

Glucose levels in plasma (Fig 1A) increased in parallel with glycaemic treatment at both stocking densities with values under HSD being higher than those under NSD under normo-, and hyper-glycaemic conditions. Plasma cortisol levels (Fig 1B) displayed a significant effect of stocking density with higher levels in the HSD groups.

**Fig 2:**
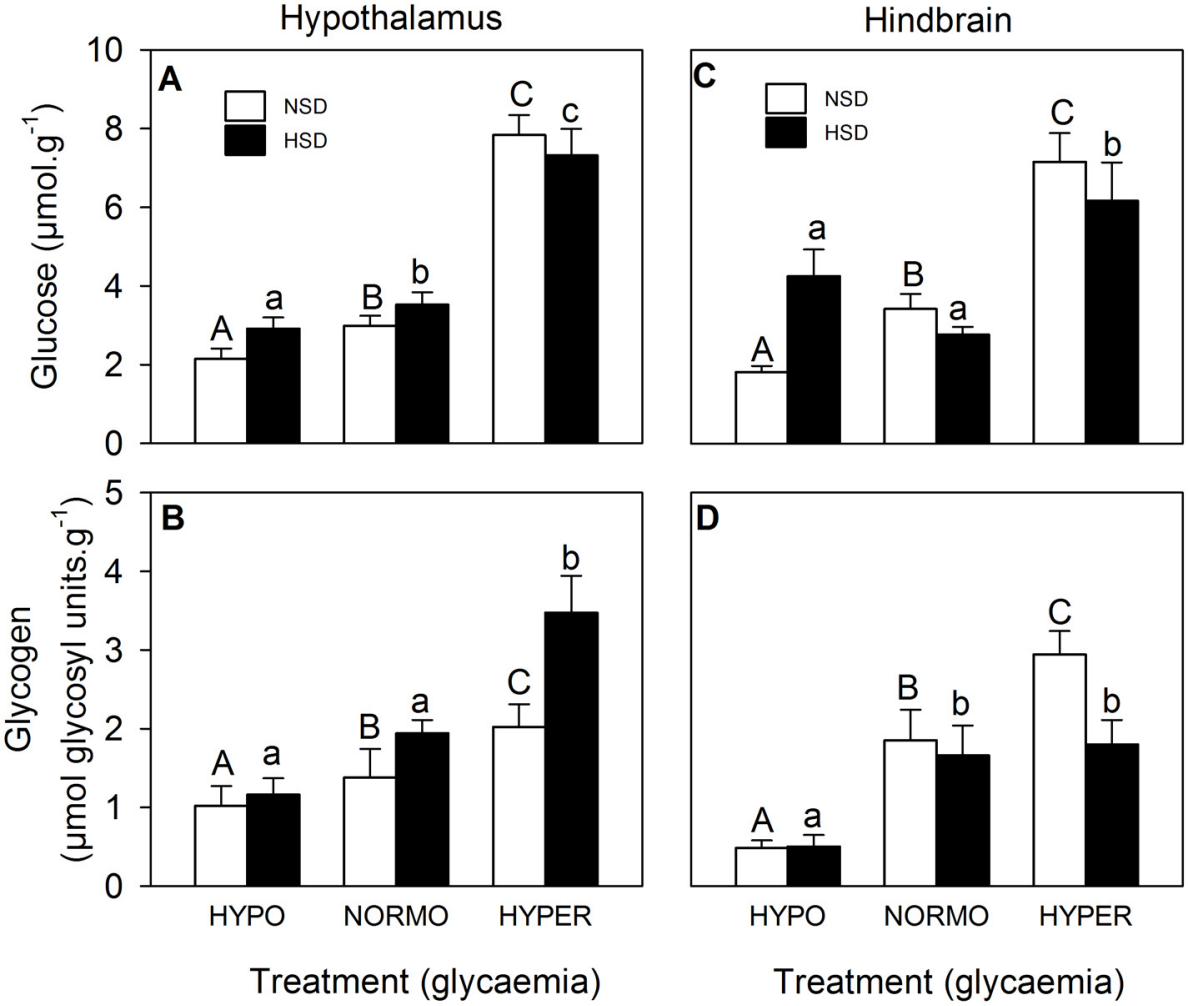
Levels of glucose (A,C) and glycogen (B,D), in hypothalamus (A,B) and hindbrain (C,D) of rainbow trout. Further details as in legend of Fig 1.

Glucose levels in hypothalamus (Fig 2A) increased in parallel with glycaemic treatment both under NSD and HSD conditions. Glucose levels in hindbrain (Fig 2C) increased in parallel with glycaemic treatment under NSD conditions whereas under HSD conditions levels were higher under hyper-glycaemic conditions. Glycogen levels also increased in parallel with glycaemic treatment in both areas (Fig 2B and 2D) under NSD conditions whereas under HSD conditions levels were higher in hyper-glycaemic fish in hypothalamus and lower in hypo-glycaemic fish in hindbrain.

**Fig 3:**
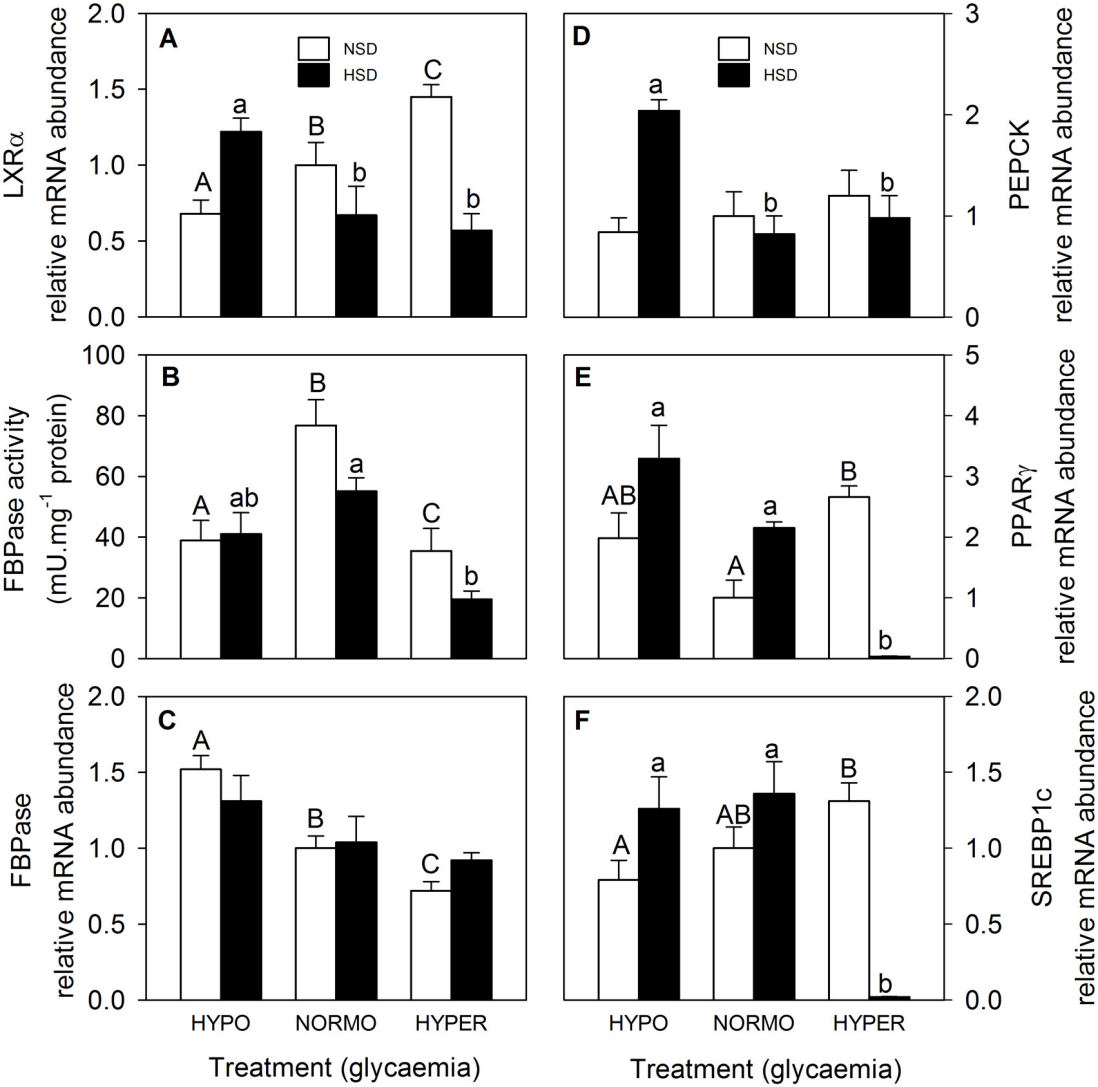
mRNA abundance of LXRα (A), FBPase (C), PEPCK (D), PPARγ (E), and SREBP1c (F), and activity of FBPase (B) in hypothalamus of rainbow trout. Data represent mean ± SEM of 5 (mRNA abundance) or 10 (enzyme activity) measurements. Data of mRNA abundance is expressed as fold-induction with respect to the normoglycaemic group kept under NSD (results were previously normalized by β-actin mRNA levels, which did not show changes among groups). *, significantly different (P<0.05) from NSD at the same glycaemic condition. Different letters indicate significant differences (P<0.05) from different glycaemic conditions at the same density (capital letters within NSD and lowercase letters within HSD).

Parameters related to glucosensing based on LXR in hypothalamus are shown in Fig 3. LXRα mRNA abundance (Fig 3A) increased in parallel with glycaemic treatment under NSD conditions whereas under HSD conditions the value of hypo-glycaemic fish was higher. FBPase activity (Fig 3B) was higher in normo-glycaemic fish than in the other glycaemic conditions for fish under NSD whereas in fish under HSD conditions the activity in hyper-glycaemic fish was higher than that of normo-glycaemic fish. The mRNA abundance of FBPase (Fig 3C) decreased in parallel with the increase in glycaemia in NSD fish with no changes being noted under HSD. PEPCK mRNA abundance (Fig 3D) did not show changes in fish under NSD whereas in fish under HSD the value of hypo-glycaemic fish was higher than those of the other glycaemic conditions. The mRNA abundance of PPARγ (Fig 3E) in fish under NSD was higher in hyper-glycaemic than in normo-glycaemic fish whereas in fish under HSD the value of hyper-glycaemic fish was lower than in the other two glycaemic conditions. Finally, SREBP1c mRNA abundance of fish under NSD increased in parallel with glycamia whereas in fish under HSD the value of hyper-glycaemic fish was lower than in the other two glycaemic conditions.

**Fig 4:**
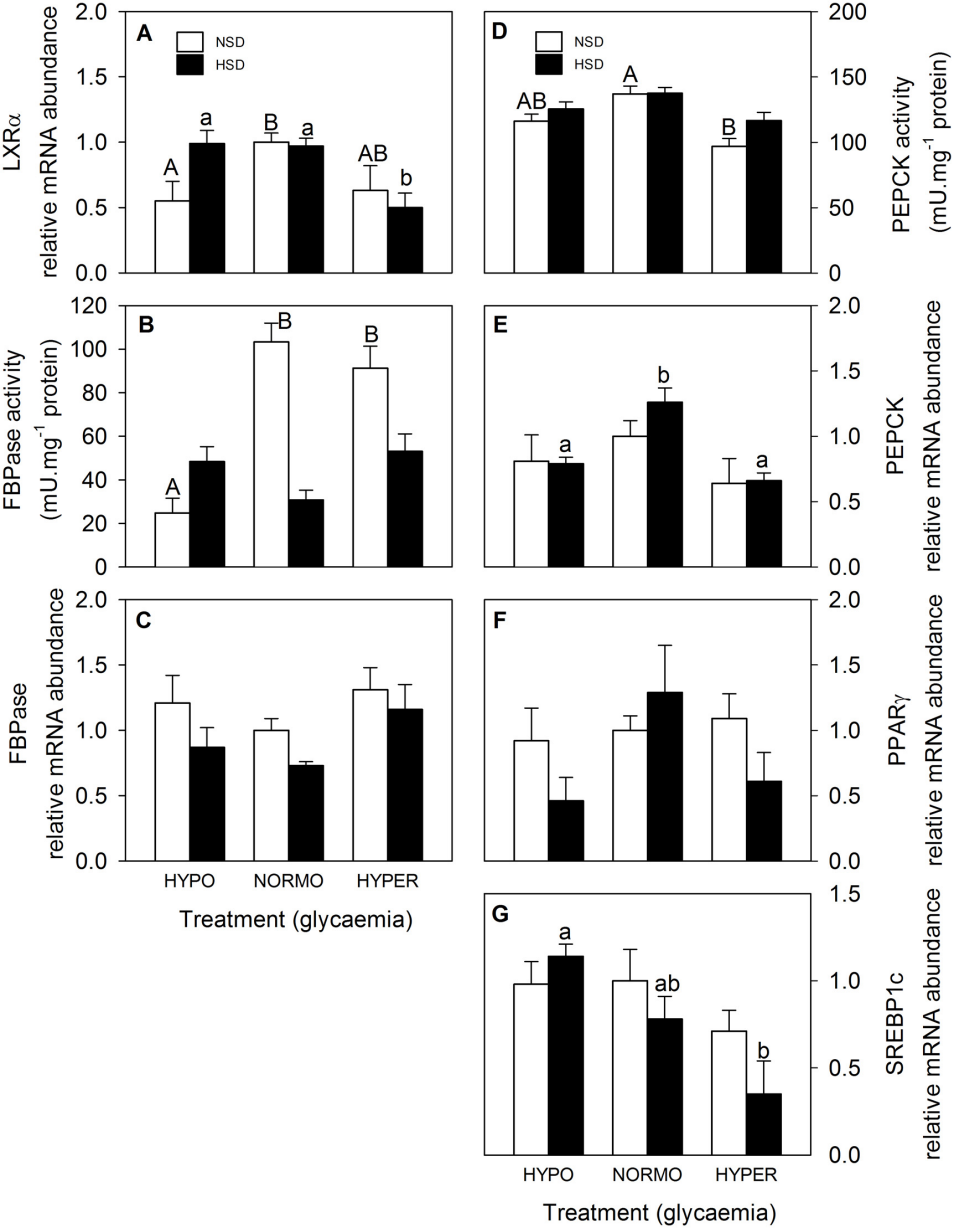
mRNA abundance of LXRα (A), FBPase (C), PEPCK (E), PPARγ (F), and SREBP1c (G), and activities of FBPase (B) and PEPCK (D) in hindbrain of rainbow trout. Further details as in legend of Fig 3.

Parameters related to glucosensing based on LXR in hindbrain are shown in Fig 4. LXRα mRNA abundance (Fig 4) in fish under NSD was lower in hypo-glycaemic than in normo-glycaemic fish whereas in fish under HSD the value of hyperglycaemic fish was lower than in the other two glycaemic conditions. FBPase activity (Fig 4B) displayed no changes in NSD fish whereas in HSD the value of hypo-glycaemic fish was lower than those of normo-and hyper-glycaemic fish. PEPCK activity (Fig 4D) in fish under NSD was lower in hyper-glycaemic than in normo-glycaemic fish whereas no changes were noted in fish under HSD. The mRNA abundance of PEPCK (Fig 4E) increased in normo-glycaemic fish compared with the two other glycaemic conditions in fish under HSD while no changes were noted for fish under NSD. The mRNA abundance of SREBP1c decreased with the increase in glycaemia in fish under HSD whereas no changes were noted in fish under NSD. Finally, no significant changes were noted for FBPase (Fig 4C) and PPARγ (Fig 4F) mRNA abundance.

**Fig 5:**
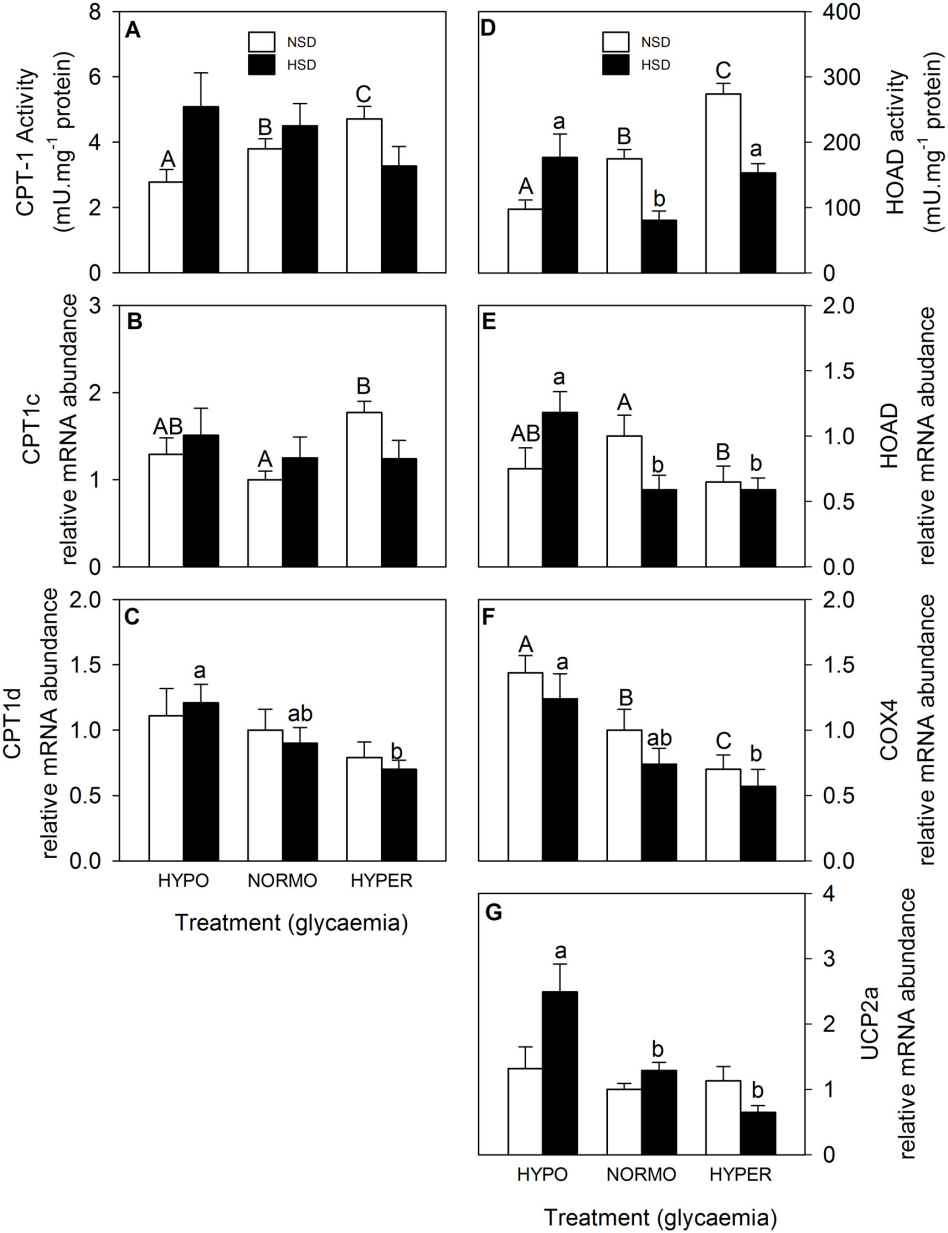
Activities of CPT-1 (A) and HOAD (D), and mRNA abundance of CPT1c (B), CPT1d (C), HOAD (E), COX4 (F), and UCP2a (G) in hypothalamus of rainbow trout. Further details as in legend of Fig 3.

The parameters associated with mitochondrial activity in hypothalamus are shown in Fig 5. CPT-1 activity (Fig 5A) increased in parallel with glycaemia in fish under NSD but no changes were noted in fish under HSD. The mRNA abundance of CPT1c (Fig 5B) in fish under NSD was higher in hyper-glycaemic than in normo-glycaemic fish whereas no changes were noted in fish under HSD. The mRNA abundance of CPT1d (Fig 5C) decreased with the increase in glycaemia in fish under HSD whereas no changes were noted for fish under NSD. HOAD activity (Fig 5D) increased in parallel with the increase in glycaemia in fish under NSD whereas in fish under HSD the value of normo-glycaemic fish was lower than in the other two glycaemic conditions. The mRNA abundance of HOAD (Fig 5E) in hyper-glycaemic fish was lower than that of normo-glycaemic fish under NSD conditions whereas in fish under HSD the value of normo-glycaemic fish was higher than in the other two glycaemic conditions. The mRNA abundance of COX4 (Fig 5F) decreased in parallel with the increase in glycaemia both under NSD and HSD conditions. Finally, the mRNA abundance of UCP2a decreased in parallel with the increase in glycaemia in fish under HSD while no changes were noted in fish under NSD.

**Fig 6:**
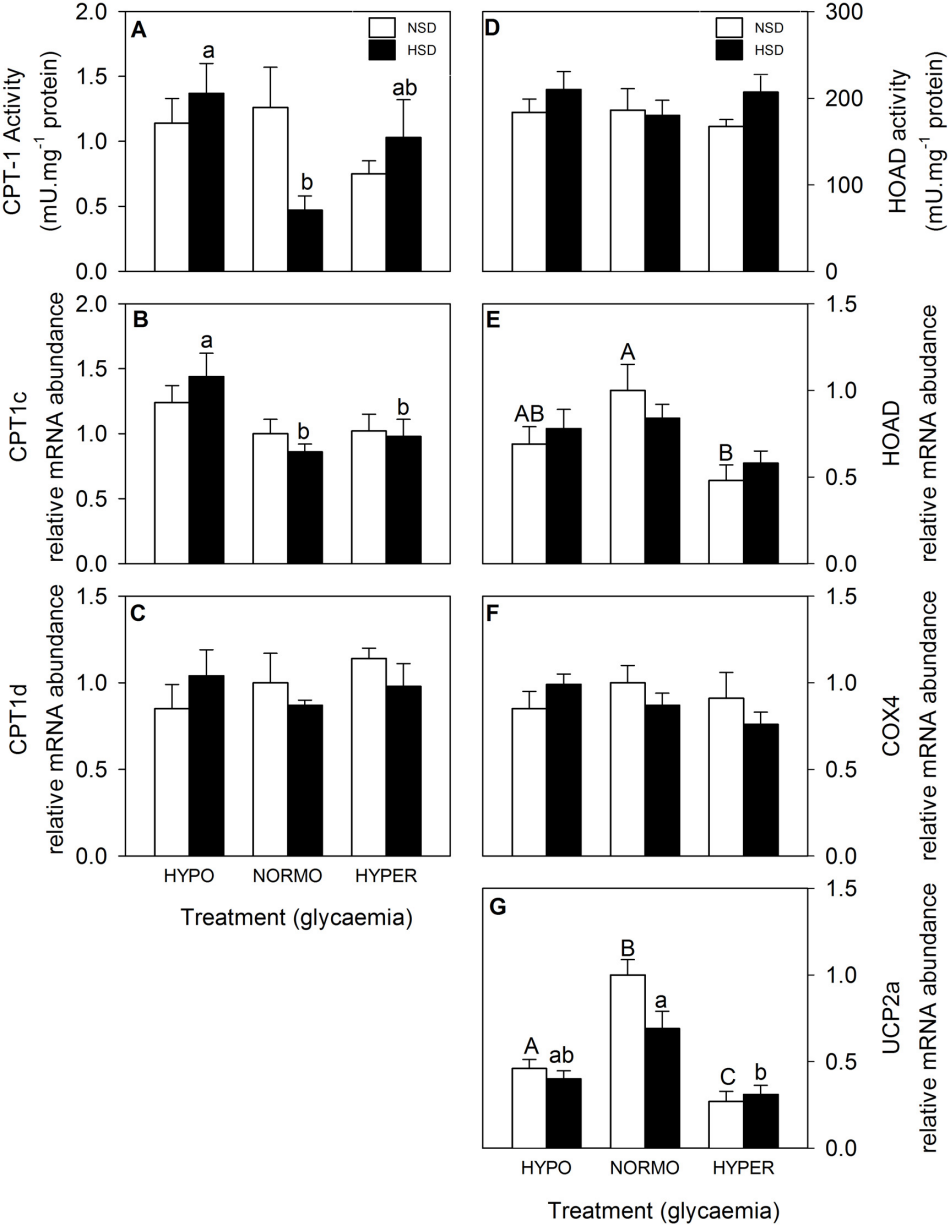
Activities of CPT-1 (A) and HOAD (D), and mRNA abundance of CPT1c (B), CPT1d (C), HOAD (E), COX4 (F), and UCP2a (G) in hindbrain of rainbow trout. Further details as in legend of Fig 3.

The parameters associated with mitochondrial activity in hindbrain are shown in Fig 6. CPT-1 activity (Fig 6A) in normo-glycaemic fish was lower than that of hypo-glycaemic fish under HSD conditions whereas no changes were noted for fish under NSD. The mRNA abundance of CPT1c (Fig 6B) of hypo-glycaemic fish was higher than in the other two glycaemic conditions for fish under HSD while no changes occurred under NSD. The mRNA abundance of HOAD (Fig 6E) was lower in hyper-glycaemic than in normo-glycaemic fish under NSD conditions; no changes were noted under HSD. The mRNA abundance of UCP2a (Fig 6G) was higher in normo-glycaemic fish than in the other two conditions in fish under NSD whereas in fish under HSD the value was higher than that of hyper-glycaemic fish. Finally, no significant changes were noted for mRNA abundance of CPT1d (Fig 6C), HOAD activity (Fig 6D), and mRNA abundance of COX4 (Fig 6F).

**Fig 7:**
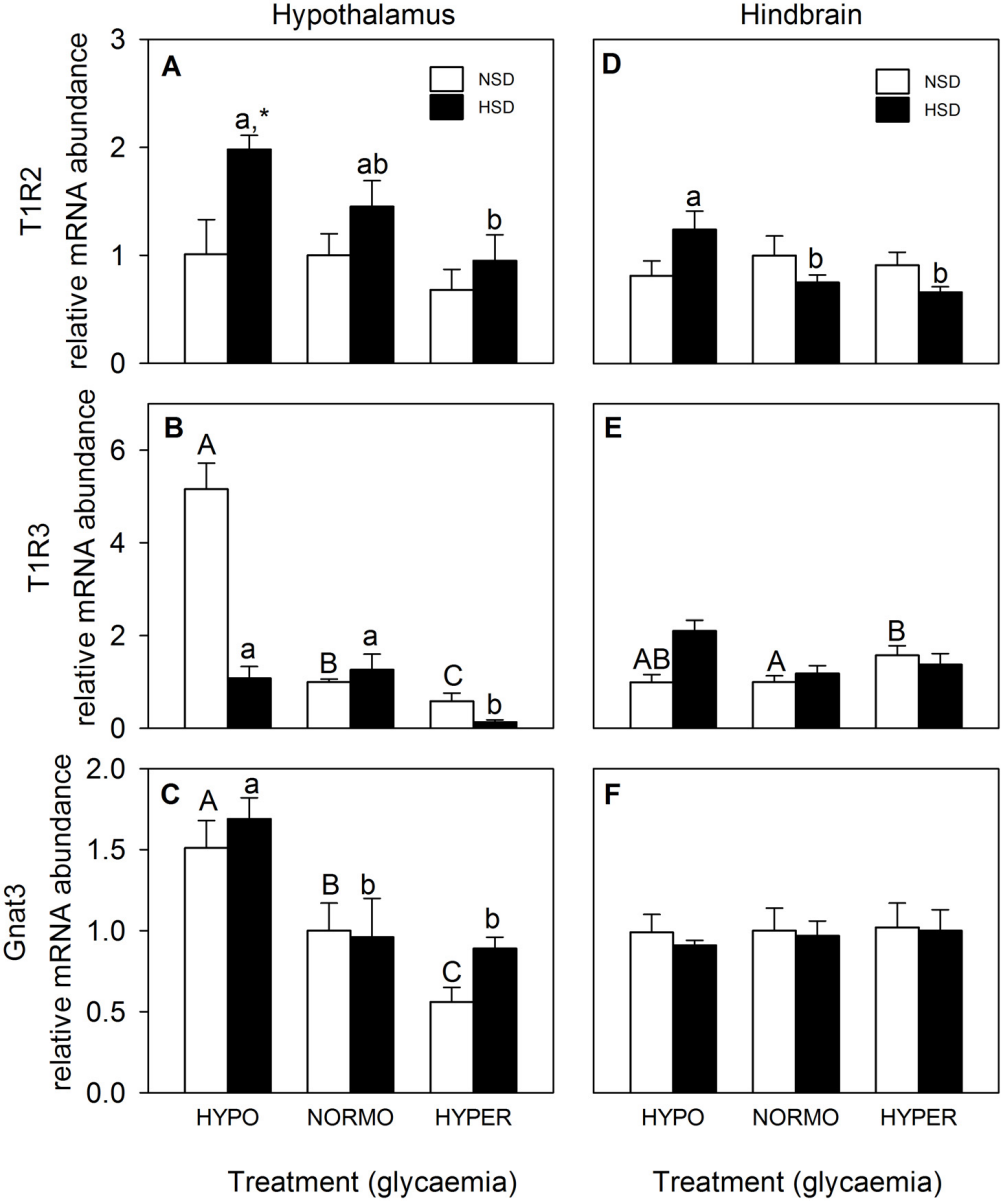
mRNA abundance of T1R2 (A,D), T1R3 (B,E), and Gnat3 (C,F) in hypothalamus (A,B,C) and hindbrain (D,E,F) of rainbow trout. Further details as in legend of Fig 3.

The parameters related to the glucosensing mechanism based on taste receptor in hypothalamus and hindbrain are shown in Fig 7. The mRNA abundance of T1R2 in hypothalamus (Fig 7A) decreased in parallel with the increase in glycaemia in fish under HSD whereas no changes were noted in fish under NSD; the value of hypo-glycaemic fish under HSD was higher than that of fish under NSD. The mRNA abundance of T1R3 (Fig 7B) decreased in parallel with the increase in glycaemia in fish under NSD whereas in fish under HSD the value of hyper-glycaemic fish is lower than in the other two glycaemic conditions. The mRNA abundance of Gnat3 in hypothalamus (Fig 7C) decreased in parallel with the increase in glycaemia in fish under NSD whereas in fish under HSD the value of hypo-glycaemic fish was higher than in the other two glycaemic conditions. The mRNA abundance of T1R2 in hindbrain (Fig 7D) was higher in normo-glycaemic fish than in the two other glycaemic conditions in fish under HSD whereas no changes were noted in fish under NSD. The mRNA abundance of T1R3 in hindbrain (Fig 7E) increased in hyper-glycaemic fish compared with normo-glycaemic fish under NSD whereas no changes were noted under HSD. Finally, no significant changes were noted for mRNA abundance of Gnat3 (Fig 7F).

**Fig 8:**
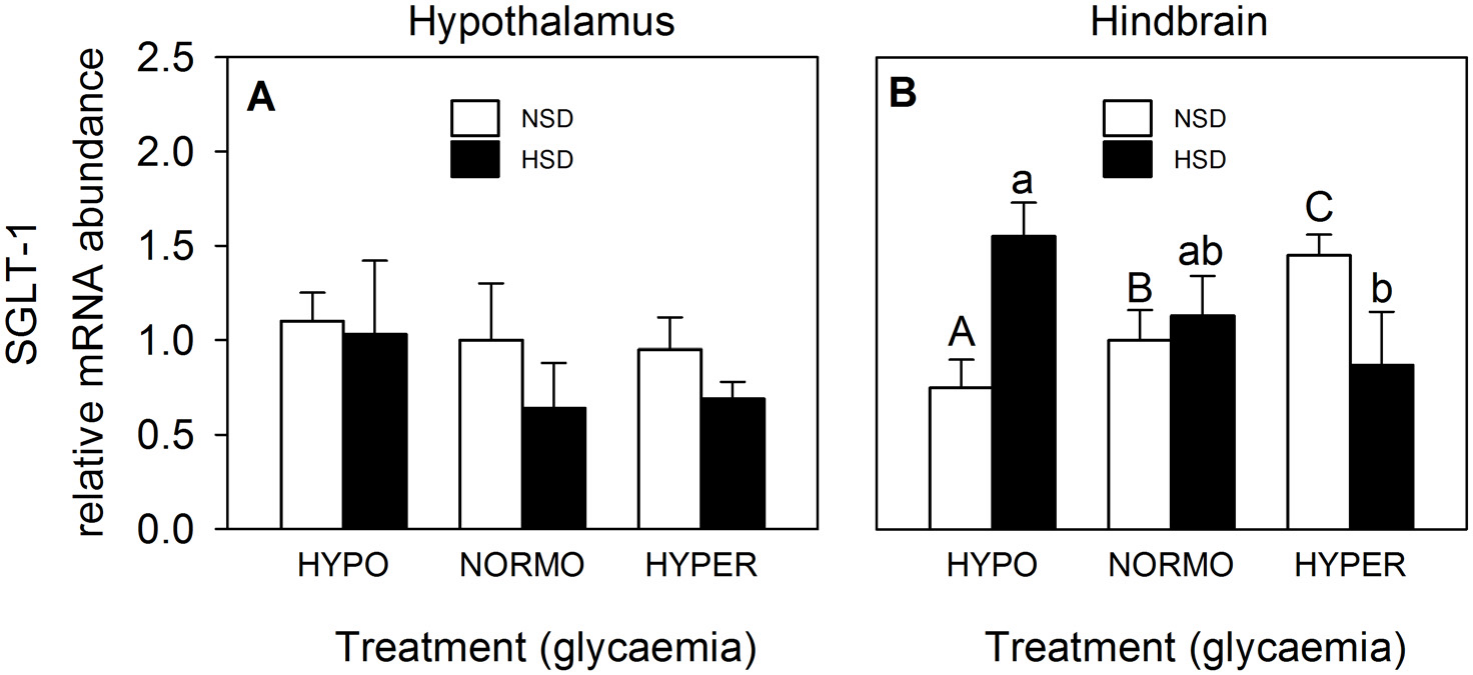
mRNA abundance of SGLT-1 in hypothalamus (A) and hindbrain (B) of rainbow trout. Further details as in legend of Fig 3.

SGLT-1 mRNA abundance was not affected by treatments in hypothalamus (Fig 8A) whereas in hindbrain (Fig 8B) values increased in parallel with glycaemia in fish under NSD while decreased under HSD.

Parameters related to glucosensing based on GK in hypothalamus are shown in S1 Fig. Significant increases in parallel with the increase in glycaemia were noted in GK activity, and mRNA abundance of GK and PK whereas a significant decrease was noted for GLUT2 mRNA abundance, and significant changes were also noted for Kir6.x-like mRNA abundance. Stocking density significantly decreased GK activity whereas significant interactions between glycaemic treatment and stocking density were noted for all parameters (except mRNA abundance of GLUT2 and Kir6.x-like).

Parameters related to glucosensing based on GK in hindbrain are shown in S2 Fig. Significant increases in parallel with the increase in glycaemia were noted in GK and PK activities, and mRNA abundance of GK, PK, and PFK whereas significant changes were also noted for Kir6.x-like mRNA abundance. Stocking density significantly affected mRNA abundance of GLUT2 and PK whereas only for GK activity a significant interaction between stocking density and glycaemic treatment was noted.

Finally, mRNA abundance of neuropeptides is shown in S3 Fig. All parameters assessed except AgRP and NPY were affected by treatments. Glycaemic treatment increased mRNA abundance of POMC-A1 and CART in hypothalamus in hyperglycaemic fish whereas levels of CART were higher in normoglycaemic fish in hindbrain. Significant interactions between glycaemic treatment and stocking density were observed in mRNA abundance of POMC-A1 and CRF in hypothalamus and CART in hindbrain.

## Discussion

### Validation of experimental design

The experimental design aimed to induce changes in circulating levels of glucose similar to those previously observed in the same species, which were able to induce a response in the glucosensing system based on GK-GLUT2-K_ATP_ [1,13,14]. Plasma levels of glucose decreased after hypo-glycaemic treatment and increased after hyper-glycaemic treatment. Similar changes occurred in glucose levels in hypothalamus and hindbrain. The different glycaemic conditions did not alter plasma cortisol levels in non-stressed fish whereas in stressed fish levels were higher than those of non-stressed fish at any glycaemic conditions. We have not used uninjected fish but levels of plasma glucose and cortisol in normo-glycaemic fish under NSD are similar to those observed in uninjected fish in previous studies thus suggesting a minor (if any) stress effect of injection. The parameters related to the glucosensor system based on GK in hypothalamus and hindbrain of fish under NSD conditions displayed changes in parallel with the increase in glycaemia (see supplementary information). These include GK activity and mRNA abundance, glycogen levels, glycolytic capacity (PK activity, and mRNA abundance of PK and PFK) and mRNA abundance of GLUT2 and components of the K_ATP_ channel. In general, these results, together with those of mRNA abundance of neuropeptides related to the control of food intake, are in agreement with those previously reported under similar experimental conditions in the same species [29,30,31,48] thus validating the experimental design.

In the next paragraphs we will discuss whether or not parameters which could be related to putative alternative glucosensing systems respond to the changes elicited in glycaemia. Since hypo-glycaemia was obtained through insulin treatment, we cannot discard that at least part of the effects observed under hypo-glycaemic conditions in all parameters assessed could be attributed to a direct insulin action.

### Glucosensing mediated by LXR

A glucosensor system based on the nuclear receptor LXR has been described in mammalian liver [5]. An increase in glucose levels activates this receptor resulting in decreased gluconeogenic capacity [6], and increased mRNA abundance of SREBP1c [49] and PPARγ [50,51]. We demonstrated in rainbow trout that LXRα mRNA abundance in intestine increased in response to elevated glucose levels either *in vivo* or *in vitro* [24] whereas in head kidney no response was noted when glucose levels were modified [26]. In the present study, a putative LXR-mediated glucosensor system could be operative in hypothalamus since increased LXRα mRNA abundance occurred in parallel with the increase in glycaemia. We have also assessed several parameters related to gluconeogenesis observing a decrease in the abundance of FBPase mRNA in parallel with the increase in glycaemia whereas a significant decrease in FBPase activity was observed when comparing normo- vs. hyper-glycaemic fish. These changes suggest an effective inhibition of gluconeogenic capacity in response to increased glucose in a way similar to that reported in mammalian liver [4,52] and to the decreased PEPCK mRNA abundance reported in zebrafish exposed to glucose [53]. Furthermore, we also observed clear changes in the mRNA abundance of transcription factors related to LXR activity such as SREBP1c and PPARγ [50,51] that displayed changes in parallel with those of glucose levels giving further support to the presence and functioning of a LXR-based glucosensing system in rainbow trout hypothalamus. The increase noted in SREBP1c mRNA abundance in response to increase glucose levels is similar to that characterized in mammalian liver [6], and in rainbow trout fed a diet rich in carbohydrates [23] and could be related to the activation of LXR as demonstrated previously in Atlantic salmon liver [19].

In contrast, in hindbrain the few changes observed in response to increased levels of glucose were not in agreement with those expected for a glucosensor system based on LXR since for instance FBPase activity increased, instead of the expected decrease resulting from decreased gluconeogenic potential.

### Glucosensing mediated by mitochondrial activity

Another metabolism-dependent but ATP-independent mechanism suggested to contribute to hypothalamic sensing of glucose and fatty acid in mammals relies on enhanced mitochondrial production of ROS by electron leakage, which is buffered by UCP2a [2,54] in a way that glucose sensing is negatively regulated by raised UCP2 activity [55,56].

In the present study, we observed in hypothalamus, but not in hindbrain, a clear increase in the activity of CPT-1 and HOAD in parallel with the increased glucose. The increased activity of both enzymes is clearly suggesting an enhanced oxidative capacity in the mitochondria in response to enhanced glucose levels. These changes are also comparable with increased mRNA abundance of CPT-1 observed in liver of rainbow trout fed with a diet rich in carbohydrates [23]. Moreover, we also observed changes in the mRNA abundance of parameters which could be related to mitochondrial activity, and these include a clear decrease in the mRNA abundance of COX4 in parallel with the increase in glucose as well as a decrease in HOAD mRNA abundance, and an increase in CPT1c when comparing hyperglycaemic vs. normo-glycaemic fish. These changes suggest the existence of enhanced mitochondrial activity in response to increased glucose, which based on the mammalian model would presumably result in enhanced ROS production. However, we did not observe the expected increase in UCP2a mRNA abundance. This is comparable to the lack of changes also noted in UCP2a mRNA abundance in hypothalamus of another fish species (orange-spotted grouper) under postprandial conditions [57] when a rise in metabolite levels (including glucose) is expected, as well as with the lack of changes observed in rainbow trout hepatocytes exposed to glucose [17]. As a whole, these results provide only partial support to the presence of a putative glucosensor system based on mitochondrial activity in trout hypothalamus. However, we cannot discard the possibility that changes in mitochondrial activity may be also dependent on the GK glucose sensing mechanism.

### Glucosensing mediated by sweet taste receptor

The transduction of the sweet taste relates to the detection of sugars including glucose in a heterodimer complex formed by T1R2+T1R3 and α-gustducin. This complex is present not only in lingual areas in mammals but also in other areas such as intestine [58], pancreatic β-cells [59] or hypothalamus [7] where is involved in glucosensing. In fish, we previously demonstrated in rainbow trout the presence of components of this system in both intestine [24] and head kidney [26]. However, only in intestine [24] a response of the evaluated parameters to changes in glucose levels was evident.

In the present study, we observed in hypothalamus, in parallel with the increase in glucose levels, a clear decrease in the mRNA abundance of T1R3 and Gnat3. This change is comparable to that already demonstrated in mammalian pancreatic β-cells [60] under similar conditions as well to that also observed in rainbow trout intestine [24]. However, rat hypothalamus responds to increased glucose levels *in vitro* with decreased T1R2 mRNA abundance but without changes in T1R3 and Gnat3 mRNA abundance [7]. In contrast, in rainbow trout, we observed decreased levels of T1R3 and Gnat3 mRNA abundance in both hypothalamus (this study) and intestine [24]. Therefore, we may suggest the existence of a different model of response of transcript abundance when comparing fish and mammals concerning glucosensing based on sweet taste receptor. In contrast, in hindbrain these changes were not observed and even an increase under hyper-glycaemic conditions was observed in that brain area.

### Glucosensing mediated by SGLT-1

SGLT-1 may act as a glucosensor by conveying information to the cell about external glucose concentration, directly through the membrane potential or indirectly coupled through a G protein [9]. Thus, the increase in glucose levels induce increased expression of SGLT-1 in different tissues of mammals thus functioning as an effective glucosensor [61]. In fish, there is evidence for the presence of this carrier in different tissues [24,25,26,62] but only in intestine there was evidence for its possible role a as glucosensor since its expression enhanced in response to increased levels of glucose [24]. In the present study, we did not observe any change in mRNA abundance of SGLT-1 in hypothalamus whereas a clear increase in hindbrain occurred in parallel with the increase in glucose. Therefore, these results suggest that SGLT-1 in hindbrain is modulated by glucose levels.

### Stress induced by high stocking density modifies the response of glucosensing parameters

Stress induced by HSD resulted in increased levels of cortisol and glucose without inducing any significant change in normo-glycaemic fish. A similar lack of effects of stress on parameters related to glucosensing based on GK was previously observed in rainbow trout under similar experimental conditions [29]. In hypothalamus, the glucosensor system based on GK did not respond to changes in glucose levels in stressed fish (see supplementary information). These results are similar to those previously reported in the same tissue and species [28,29] and are in agreement with the results observed in the expression of neuropeptides.

In the parameters which could be related to putative glucosensor system based on LXR, mitochondrial activity and sweet taste receptor in hypothalamus, and SGLT-1 in hindbrain, an interaction between glycaemic conditions and stress induced by HSD was noted in several parameters. Thus, the response of these parameters to changes in glucose levels is altered under the stress conditions elicited by high stocking density when compared with non-stressed fish. The precise connection between stress and the function of alternative glucosensing mechanisms has to be stablished in further studies. However, the effects of stress on the systems could relate to the action of any of the components of the hypothalamus-pituitary-interrenal (HPI) axis, such as CRF whose mRNA abundance displayed changes in hypothalamus of stressed fish depending on glycaemia, as demonstrated previously in the modulation by CRF of the glucosensor system based on GK in rainbow trout hypothalamus [63].

## Conclusions

The results obtained in non-stressed rainbow trout evidence for the first time in fish, and in a non-mammalian vertebrate, that manipulation of glucose levels affects parameters which could be related to putative glucosensor systems based on LXR, mitochondrial activity, and sweet taste receptor in hypothalamus and on SGLT-1 in hindbrain. We cannot discard, however, that some of the changes may be also due to GK activation. This differential response between brain areas is different than that of GK-mediated system, which responded to glucose similarly in both brain areas [1,13,14], and to that known in the mammalian model [1]. Despite rainbow trout is a carnivorous species, the responses observed for alternative glucosensing systems in hypothalamus are more comparable in general to those described in omnivorous mammals [3] in the same area than to those observed in peripheral areas, like intestine, in the few available studies in carnivorous mammals like cats [12]. The stress conditions elicited by HSD altered the response to glucose of parameters that could be associated with putative glucosensor systems in hypothalamus and hindbrain. Further studies are necessary to characterize the underlying mechanisms and the physiological role (control of food intake, counter-regulatory mechanisms) of these putative alternative glucosensing capabilities of rainbow trout brain.

## Supporting Information

S1 Table P-values obtained after two-way analysis of variance. Parameters related to GK-mediated glucosensing were assessed in rainbow trout under different glycaemic conditions elicited by intraperitoneal (IP) administration of saline solution alone (normoglycaemic) or containing insulin (hypoglycaemic, 4 mg bovine insulin.Kg^-1^ body mass), or D-glucose (hyperglycaemic, 500 mg.Kg^-1^ body mass) kept at normal stocking density (NSD, 10 kg.m^-3^) or high stocking density (HSD, 70 kg.m^-3^) for 6 hours. Glycaemia (hypo-, normo-, and hyper-) and stocking density (NSD and HSD) were the main factors. All values are significantly different unless noted by a dash.
(DOCX)

S1 Fig Parameters related to glucosensing based on GK in hypothalamus. mRNA abundance of GLUT2 (A), GK (C), PK (D), PFK (E), Kir6.x-like (F) and SUR-like (G) and activity of GK (B) in hypothalamus of rainbow trout. Data represent mean ± SEM of 5 (mRNA abundance) or 10 (enzyme activity) measurements. Data of mRNA abundance is expressed as fold-induction with respect to the normoglycaemic group kept under NSD (results were previously normalized by β-actin mRNA levels, which did not show changes among groups). *, significantly different (P<0.05) from NSD at the same glycaemic condition. Different letters indicate significant differences (P<0.05) from different glycaemic conditions at the same density (capital letters within NSD and lowercase letters within HSD).
(TIF)

S2 Fig Parameters related to glucosensing based on GK in hindbrain. mRNA abundance of GLUT2 (A), GK (C), PK (E), PFK (F), Kir6.x-like (G) and SUR-like (H) and activities of GK (B) and PK (D) in hindbrain of rainbow trout. Data represent mean ± SEM of 5 (mRNA abundance) or 10 (enzyme activity) measurements. Data of mRNA abundance is expressed as fold-induction with respect to the normoglycaemic group kept under NSD (results were previously normalized by β-actin mRNA levels, which did not show changes among groups). *, significantly different (P<0.05) from NSD at the same glycaemic condition. Different letters indicate significant differences (P<0.05) from different glycaemic conditions at the same density (capital letters within NSD and lowercase letters within HSD).
(TIF)

S3 Fig Neuropeptide expression. mRNA abundance of AgRP (A,F), NPY (B,G), POMC-A1 (C,H), CART (D,I), and CRF (E,J) in hypothalamus (A,B,C,D,E) and hindbrain (F,G,H,I,J) of rainbow trout. Data represent mean ± SEM of 5 measurements. Data of mRNA abundance is expressed as fold-induction with respect to the normoglycaemic group kept under NSD (results were previously normalized by β-actin mRNA levels, which did not show changes among groups). *, significantly different (P<0.05) from NSD at the same glycaemic condition. Different letters indicate significant differences (P<0.05) from different glycaemic conditions at the same density (capital letters within NSD and lowercase letters within HSD).
(TIF)
