# Supplementary material for: Evidence for the Presence of Glucosensor Mechanisms Not Dependent on Glucokinase in Hypothalamus and Hindbrain of Rainbow Trout (Oncorhynchus mykiss)
Source: PLoS One. 2015 May 21;10(5):e0128603. doi: 10.1371/journal.pone.0128603 (PMC4440750; doi:10.1371/journal.pone.0128603)
Supplement: S1 Table — Parameters related to GK-mediated glucosensing were assessed in rainbow trout under different glycaemic conditions elicited by intraperitoneal (IP) administration of saline solution alone (normoglycaemic) or containing insulin (hypoglycaemic, 4 mg bovine insulin.Kg-1 body mass), or D-glucose (hyperglycaemic, 500 mg.Kg-1 body mass) kept at normal stocking density (NSD, 10 kg.m-3) or high stocking density (HSD, 70 kg.m-3) for 6 hours. Glycaemia (hypo-, normo-, and hyper-) and stocking density (NSD and HSD) were the main factors. All values are significantly different unless noted by a dash. (DOCX) [file pone.0128603.s001.docx]

| Parameter | Glycaemia | Stocking density | Glycaemia x stocking density |
| --- | --- | --- | --- |
| Hypothalamus |  |  |  |
| GLUT2 mRNA abundance | 0.039 | - | - |
| GK activity | 0.042 | 0.044 | 0.041 |
| GK mRNA abundance | 0.046 | - | 0.045 |
| PK mRNA abundance | 0.001 | - | 0.048 |
| PFK mRNA abundance | 0.049 | - | 0.036 |
| Kir6.x-like mRNA abundance | 0.001 | - | - |
| SUR-like mRNA abundance | 0.033 | - | 0.039 |
| AgRP mRNA abundance | - | - | 0.023 |
| NPY mRNA abundance | - | - | - |
| POMC-A1 mRNA abundance | 0.041 | - | 0.029 |
| CART mRNA abundance | 0.031 | - | - |
| CRF mRNA abundance | 0.038 | - | 0.034 |
| Hindbrain |  |  |  |
| GLUT2 mRNA abundance | 0.001 | 0.024 | - |
| GK activity | 0.015 | - | 0.032 |
| GK mRNA abundance | 0.029 | - | - |
| PK activity | 0.042 | - | - |
| PK mRNA abundance | 0.027 | 0.015 | - |
| PFK mRNA abundance | 0.003 | - | - |
| Kir6.x-like mRNA abundance | 0.005 | - | - |
| SUR-like mRNA abundance | - | - | - |
| AgRP mRNA abundance | - | - | - |
| NPY mRNA abundance | - | - | - |
| POMC-A1 mRNA abundance | - | 0.043 | - |
| CART mRNA abundance | 0.041 | - | 0.021 |
| CRF mRNA abundance | 0.023 | - | - |
