# Supplementary figures and images for: Evidence for the Presence of Glucosensor Mechanisms Not Dependent on Glucokinase in Hypothalamus and Hindbrain of Rainbow Trout (Oncorhynchus mykiss)
Source: PLoS One. 2015 May 21;10(5):e0128603. doi: 10.1371/journal.pone.0128603 (PMC4440750; doi:10.1371/journal.pone.0128603)

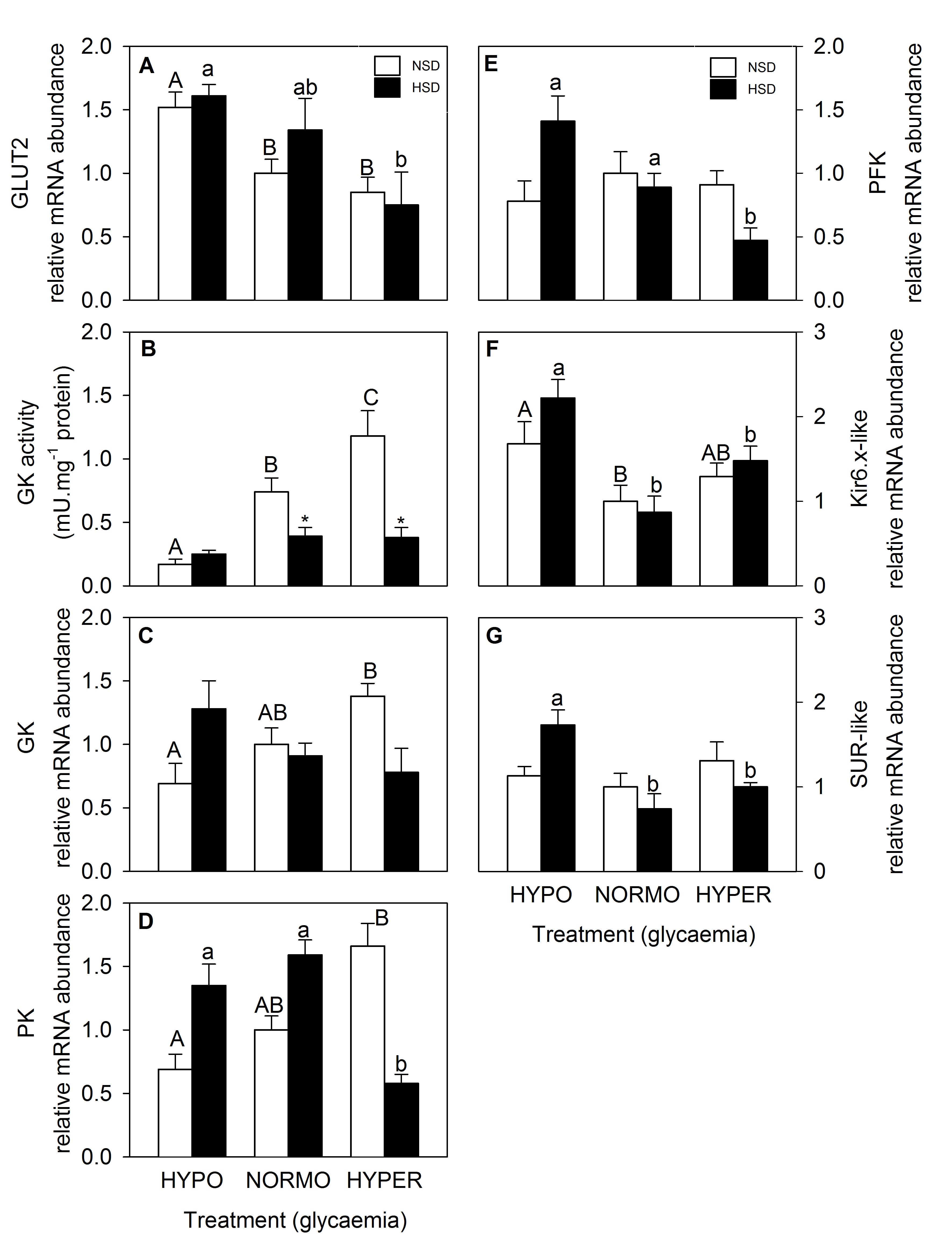

Supplement: S1 Fig — mRNA abundance of GLUT2 (A), GK (C), PK (D), PFK (E), Kir6.x-like (F) and SUR-like (G) and activity of GK (B) in hypothalamus of rainbow trout. Data represent mean ± SEM of 5 (mRNA abundance) or 10 (enzyme activity) measurements. Data of mRNA abundance is expressed as fold-induction with respect to the normoglycaemic group kept under NSD (results were previously normalized by β-actin mRNA levels, which did not show changes among groups). *, significantly different (P<0.05) from NSD at the same glycaemic condition. Different letters indicate significant differences (P<0.05) from different glycaemic conditions at the same density (capital letters within NSD and lowercase letters within HSD). (TIF) [file pone.0128603.s002.TIF]

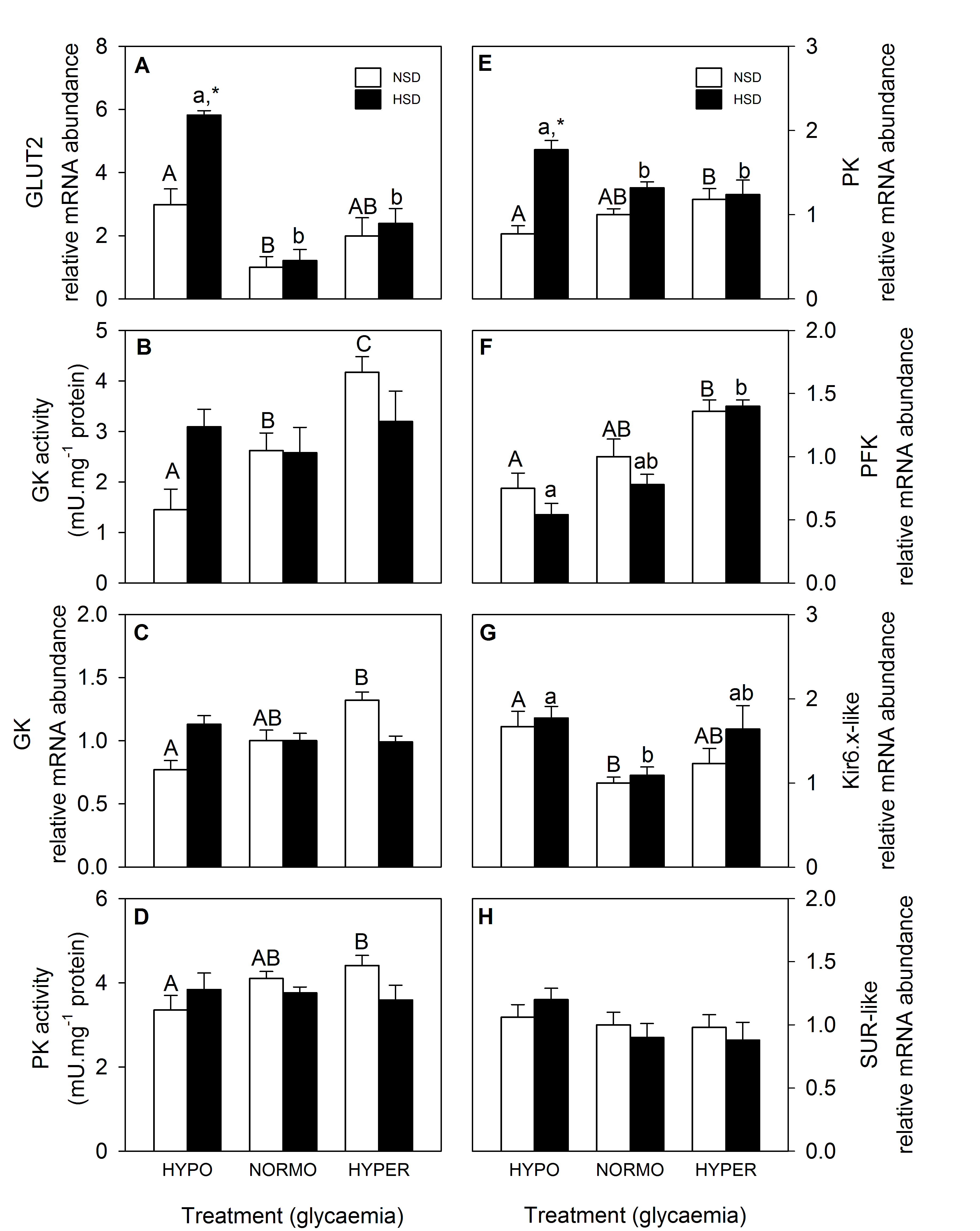

Supplement: S2 Fig — mRNA abundance of GLUT2 (A), GK (C), PK (E), PFK (F), Kir6.x-like (G) and SUR-like (H) and activities of GK (B) and PK (D) in hindbrain of rainbow trout. Data represent mean ± SEM of 5 (mRNA abundance) or 10 (enzyme activity) measurements. Data of mRNA abundance is expressed as fold-induction with respect to the normoglycaemic group kept under NSD (results were previously normalized by β-actin mRNA levels, which did not show changes among groups). *, significantly different (P<0.05) from NSD at the same glycaemic condition. Different letters indicate significant differences (P<0.05) from different glycaemic conditions at the same density (capital letters within NSD and lowercase letters within HSD). (TIF) [file pone.0128603.s003.TIF]

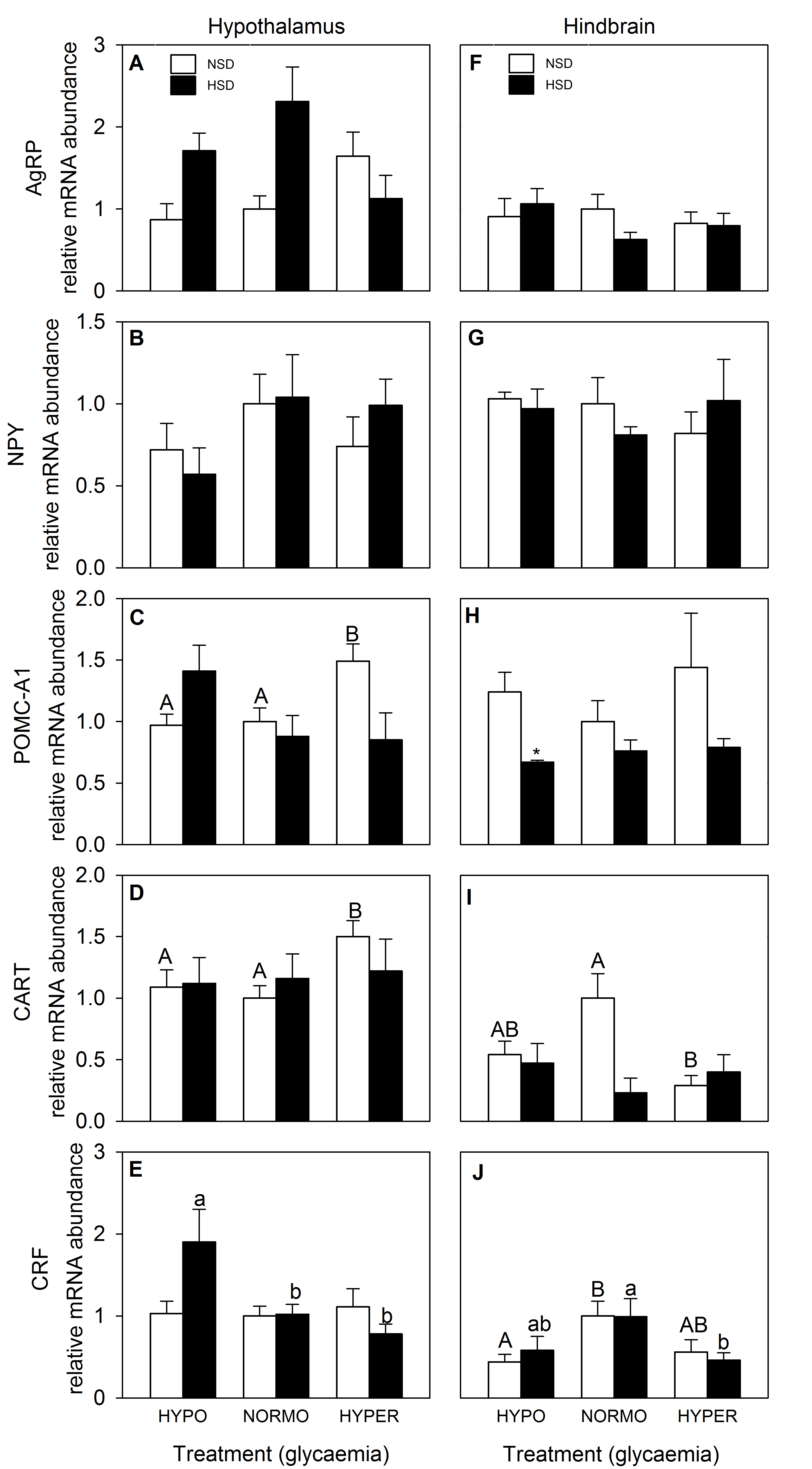

Supplement: S3 Fig — mRNA abundance of AgRP (A,F), NPY (B,G), POMC-A1 (C,H), CART (D,I), and CRF (E,J) in hypothalamus (A,B,C,D,E) and hindbrain (F,G,H,I,J) of rainbow trout. Data represent mean ± SEM of 5 measurements. Data of mRNA abundance is expressed as fold-induction with respect to the normoglycaemic group kept under NSD (results were previously normalized by β-actin mRNA levels, which did not show changes among groups). *, significantly different (P<0.05) from NSD at the same glycaemic condition. Different letters indicate significant differences (P<0.05) from different glycaemic conditions at the same density (capital letters within NSD and lowercase letters within HSD). (TIF) [file pone.0128603.s004.TIF]
